# Testing early warning and response systems through a full-scale exercise in Vietnam

**DOI:** 10.1186/s12889-021-10402-x

**Published:** 2021-02-26

**Authors:** Alexey Clara, Anh T. P. Dao, Quy Tran, Phu D. Tran, Tan Q. Dang, Huong T. Nguyen, Quang D. Tran, Peter Rzeszotarski, Karen Talbert, Tasha Stehling-Ariza, Frances Veasey, Lynne Clemens, Anthony W. Mounts, Hannah Lofgren, S. Arunmozhi Balajee, Trang T. Do

**Affiliations:** 1 Division of Viral Diseases, National Center for Immunization and Respiratory Diseases, Centers for Disease Control and Prevention, Atlanta, GA USA; 2 Division of Global Health Protection, Center for Global Health, Centers for Disease Control and Prevention, Atlanta, USA; 3 General Department of Preventive Medicine, Under the Vietnam Ministry of Health, Hanoi, Vietnam; 4 Division of Emergency Operations, Center for Preparedness and Response, Centers for Disease Control and Prevention, Atlanta, USA; 5 Analytic Services, Inc (ANSER), Virginia, USA

**Keywords:** Early warning and response, Full scale exercise, Vietnam

## Abstract

**Background:**

Simulation exercises can functionally validate World Health Organization (WHO) International Health Regulations (IHR 2005) core capacities. In 2018, the Vietnam Ministry of Health (MOH) conducted a full-scale exercise (FSX) in response to cases of severe viral pneumonia with subsequent laboratory confirmation for Middle East Respiratory Syndrome Coronavirus (MERS-CoV) to evaluate the country’s early warning and response capabilities for high-risk events.

**Methods:**

An exercise planning team designed a complex fictitious scenario beginning with one case of severe viral pneumonia presenting at the hospital level and developed all the materials required for the exercise. Actors, controllers and evaluators were trained. In August 2018, a 3-day exercise was conducted in Quang Ninh province and Hanoi city, with participation of public health partners at the community, district, province, regional and national levels. Immediate debriefings and an after-action review were conducted after all exercise activities. Participants assessed overall exercise design, conduction and usefulness.

**Results:**

FSX findings demonstrated that the event-based surveillance component of the MOH surveillance system worked optimally at different administrative levels. Detection and reporting of signals at the community and health facility levels were appropriate. Triage, verification and risk assessment were successfully implemented to identify a high-risk event and trigger timely response. The FSX identified infection control, coordination with internal and external response partners and process documentation as response challenges. Participants positively evaluated the exercise training and design.

**Conclusions:**

This exercise documents the value of exercising surveillance capabilities as part of a real-time operational scenario before facing a true emergency. The timing of this exercise and choice of disease scenario was particularly fortuitous given the subsequent appearance of COVID-19. As a result of this exercise and subsequent improvements made by the MOH, the country may have been better able to deal with the emergence of SARS-CoV-2 and contain it.

**Supplementary Information:**

The online version contains supplementary material available at 10.1186/s12889-021-10402-x.

## Background

Simulation exercises have been identified as a functional means of validating the World Health Organization’s (WHO) International Health Regulations (IHR 2005) core capacities [1–5]. They involve the practice, training, monitoring or evaluation of capabilities using the description or simulation of an emergency, to which a described or simulated response is made. Full-scale exercises, one of the four fundamental types of simulation exercises, simulate real events as closely as possible. They are designed to evaluate the operational capability of emergency management systems in a highly stressful environment, simulating actual response conditions. An FSX can test and evaluate most functions of an emergency management plan or operational plan [5, 6].

To improve national capacity to prevent, detect and respond ahead of outbreaks, and to meet obligations under the IHR 2005, the Ministry of Health (MOH) of Vietnam has been strengthening the early warning components of their surveillance system as part of the Global Health Security Agenda (GHSA). GHSA is a collaborative multisectoral effort to accelerate and optimize global health security [7–9].

Vietnam has four administrative health regions; each has a regional public health institute that is responsible for technical direction and supervision of surveillance and response to diseases and outbreaks in that region [10, 11]. Within each region, provincial centers for disease control lead surveillance and response activities in their respective provinces; in turn, district health centers within provinces coordinate district-level public health activities. Districts are divided into communes that each have a commune health station, which is the primary healthcare unit in Vietnam [12]. Within each commune, village health workers and health collaborators constitute community networks and support the commune health station in different health-promotion activities. Nationally, the General Department of Preventive Medicine (GDPM), an agency within the Ministry of Health, provides public health policy and the strategic direction of public health activities, including surveillance.

In 2013, the GDPM established a national Public Health Emergency Operation Center (PHEOC) to manage risk assessment and response to public health threats [13]. The national PHEOC conducted several tabletop exercises and drills for Middle East Respiratory Syndrome Coronavirus (MERS-CoV), Ebola, influenza A (H7N9) and influenza A (H5N1), which were useful to practice and assess functional aspects of the PHEOC during a public health emergency response [8].

Vietnam has several surveillance systems that aggregate data from a variety of sources, including communities and health facilities, which are required to routinely report notifiable diseases through the public health system from communes and districts to the province level and then the regional institutes [13].

In 2016 and 2017, the MOH launched a pilot program focused on strengthening community and hospital event-based surveillance (EBS) in six of Vietnam’s 63 provinces. Vietnam’s EBS is a system of monitoring for disease patterns in health care facilities and communities that may signal the emergence of an acute risk to human health [14, 15]. It includes ad hoc direct reporting of signals from designated officials in health care facilities and community health workers who are in contact with key informants that live in the community. A comprehensive evaluation of the EBS project was conducted after a pilot in six provinces [10, 11, 16]. Data from the evaluation were used to make improvements to the program before its subsequent national roll-out. The pilot demonstrated that EBS resulted in early detection and reporting of outbreaks, improved collaboration between the healthcare facilities and preventive sectors of the ministry and increased community participation in surveillance and reporting [10, 16].

The Vietnam MOH, in collaboration with the U.S. Centers for Disease Control and Prevention (CDC), the WHO Office in Vietnam (WHO VTN) and Analytic Services Inc. (ANSER, a non-profit organization), planned and conducted a FSX in 2018 to evaluate the country’s public health emergency capabilities for response to cases of very high-risk events by conducting simulation exercises in real-time. The FSX was designed to test the early warning and response system incorporating EBS, Rapid Response Team (RRT) activation and deployment, laboratory responsiveness and integration with epidemiological data, and PHEOC functions. The current manuscript describes the design and conduct of the FSX with a focus on early warning and response and the lessons learned from this experience.

## Methods

### Establishing an exercise planning team

An exercise planning team was established with representatives from the Vietnam MOH’s GDPM, the National Institute of Hygiene and Epidemiology (NIHE), WHO VTN, CDC and ANSER. The planning team finalized the objectives for the FSX (Table 1); consulted the Provincial Center for Diseases Control (PCDC), which had been selected as a field site for the FSX, about local contexts and feasibility of the scenario; and developed all the materials required for the successful completion of the exercise.

**Table 1:** Objectives, WHO IHR core capacities and relevant GHSA capacities addressed by the 2018 full-scale exercise in Vietnam

Objectives	WHO IHR core capacities
**At Commune Health Station** (*Quang Ninh province, Cam Pha city, Cam Dong ward*) 1. The Commune Health Station follows established protocols to triage, verify and report signals to the District Health Center. **At District Hospital ***(Quang Ninh province, Cam Pha city)* 2. The District Hospital uses hospital signal criteria to detect and report signal. 3. The District Hospital follows established protocols to collect specimens and package them for transport to the lab for testing. **At District Health Center ***(Quang Ninh province, Cam Pha city)* 4. The District Health Center follows established protocols to verify suspect cases and report public health events to the provincial level. 5. The District Health Center RRT deploys within timeframe requested in notification and performs public health emergency response activities according to established protocols **At PCDC** *(Quang Ning province)* 6. The PCDC follows established protocols to conduct disease surveillance, risk assessments and disease reporting in support of a public health emergency. 7. The PCDC coordinates appropriate risk messaging in support of a public health emergency. 8. The PCDC RRT deploys within timeframe requested in notification and performs public health emergency response activities according to established protocols 9. The PCDC lab follows established protocols to receive specimens, test specimens and report results within an appropriate amount of time. **At National Institute of Hygiene and Epidemiology ***(Hanoi city*) 10. The Regional lab follows established protocols to receive and test specimens. 11. The Regional lab reports specimen test results to the District and provincial levels within an appropriate amount of time from original receipt of the specimens. **At North Region PHEOC** *(Hanoi city*) 12. The Regional PHEOC follows established protocols to activate and establish a coordinated emergency response after identification of a public health emergency. 13. The Regional PHEOC follows established protocols to perform situational monitoring and reporting and to develop an IAP. 14. The Regional PHEOC provides support to Province to develop public health messaging and implement risk communication in response to a public health emergency. 15. The North Region RRT deploys within timeframe requested in notification and performs public health emergency response activities according to established protocols. **At National PHEOC** *(Hanoi city)* 16. The National PHEOC activates a coordinated emergency response within the required amount of time from confirmation of a public health emergency. 17. The National PHEOC follows established protocols to perform situational monitoring and reporting and to develop an IAP. 18. The National PHEOC develops public health messaging and implements risk communication within 24 h of confirmation of a public health emergency. 19. The IHR Focal Point implements established case management procedures for IHR relevant hazards during the public health emergency.	• **D.1** National Laboratory system • **D.2** Real-time surveillance • **D.3** Reporting • **R.1** Preparedness • **R.2** Emergency Response Operations • **R.4** Medical countermeasures and personnel deployment • **R.5** Risk communication
	**Relevant GHSA capacities**
	• **Detect** • **Response**

Abbreviations: *GHSA* Global Health Security Agenda, *IAP* Incident Action Plan, *IHR* International Health Regulations, *PCDC* Provincial Center for Disease Control, *PHEOC* Public Health Emergency Operations Center, *WHO* World Health Organization

### Selection of FSX sites

**Fig. 1:**
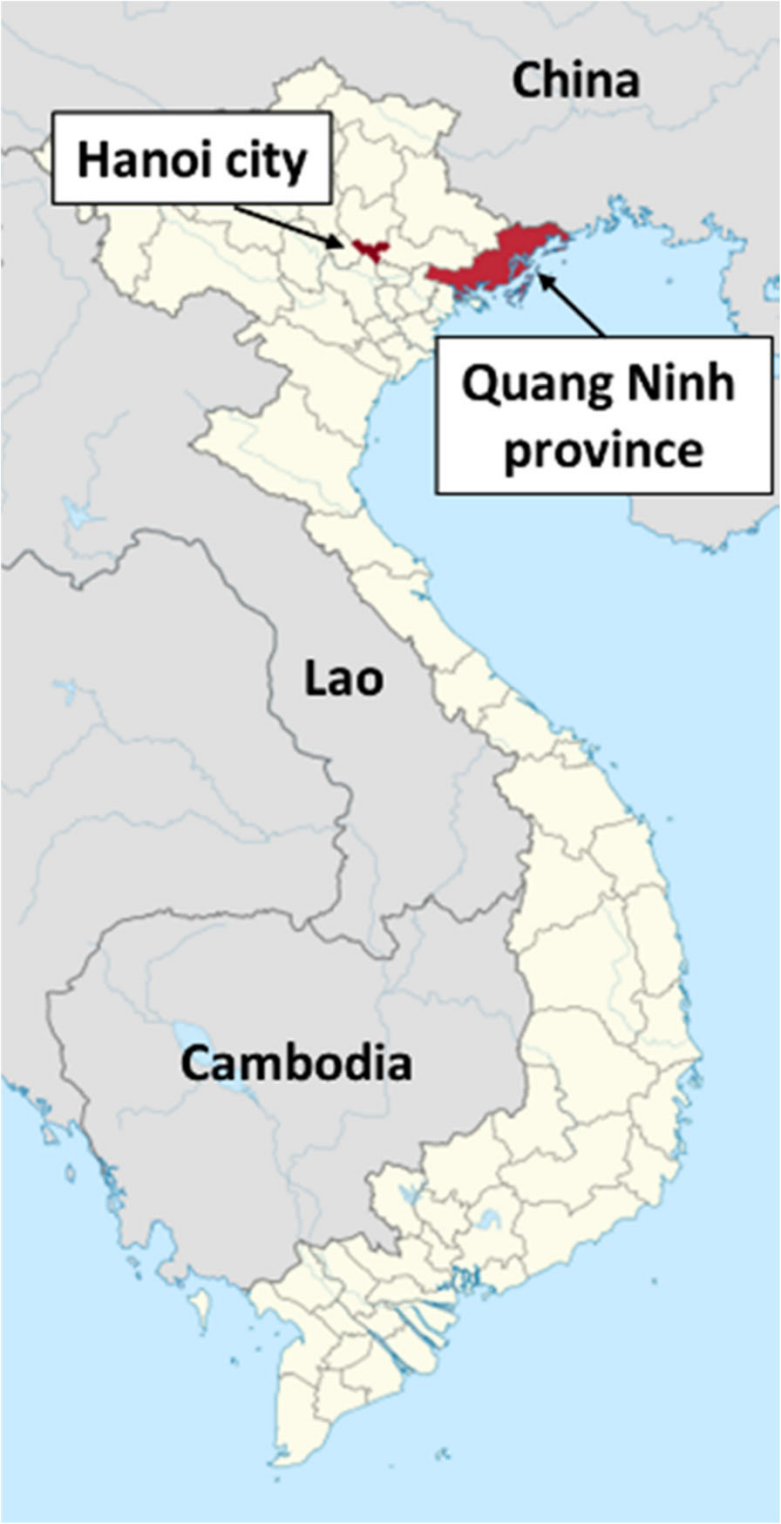
Locations for the 2018 full-scale exercise in Vietnam. The exercise was conducted in Quang Ninh province and Hanoi city (both in red). *Source: Wikimedia Commons (*https://commons.wikimedia.org/wiki/File:Vietnam_location_map.svg*)**. The original figure was slightly modified by the authors of this manuscript*

Quang Ninh province was selected for the conduct of the exercise at community, district and provincial levels, and Hanoi city, the capital of Vietnam, hosted the exercise activities at regional and national levels. Quang Ninh province is located along the northeastern coast of Vietnam, 153 km east of Hanoi, and was one of the six provinces that participated at the province, district and commune levels in the EBS pilot implementation project in 2016–2017. The Vietnam MOH Headquarters, the NIHE Regional Public Health Laboratory and National and Northern Regional Public Health Emergency Operations Centers are all located in Hanoi city (Fig. 1).

### Participants, roles and responsibilities

Seven types of participants were defined for each operational unit: players, controllers, evaluators, venue points of contact, actors, observers and media personnel (Table 2). Participants in the FSX were representatives from all levels of the MOH, CDC and WHO VTN.

**Table 2:** Participants and roles and responsibilities, 2018 full-scale exercise in Vietnam

Participants	Roles and responsibilities
Players	Players are personnel who have an active role in discussing or performing their regular roles and responsibilities involved during the exercise. Players discuss or initiate actions in response to the simulated scenario (e.g., doctors, nurses, laboratory staff).
Controllers	Controllers plan and manage exercise play, set up and operate the exercise site and act in the roles of organizations or individuals who are not playing in the exercise. Controllers direct the pace of the exercise, provide key data to Players and may prompt or initiate certain Players’ actions to ensure exercise continuity. In addition, they issue material to Players as required, monitor the exercise timeline and supervise the safety of all exercise participants. There were 3 types of controllers: exercise director, exercise lead controller (control cell) and venue controller.
Evaluators	Evaluators observe exercise events and provide feedback on designated functional areas. Evaluators document performance against established capability targets and critical tasks. Ten evaluators participated in the full-scale exercise.
Venue Points of Contact	Venue Points of Contact are trusted agents at participating organizations who are aware of the exercise and can assist the Venue Controllers. These individuals are trusted within the organization and can be relied upon to provide official verification that the exercise is authorized, and that the participants should cooperate with the Venue Controller.
Actors	Actors simulate specific roles during exercise play, typically victims or other bystanders. Actors simulated patients, family members and contacts during exercise play (e.g., businessman, his wife, driver and household employee as described in the exercise scenario).
Observers	Observers visit or view selected segments of the exercise. Observers do not perform in the exercise, nor do they perform any control or evaluation functions. Observers view the exercise from a designated observation area and must remain within the observation area during the exercise.
Media Personnel	Some media personnel may be present as observers, pending approval by the sponsor organization and the Exercise Planning Team.

### Materials developed for the exercise

The planning team developed the following materials for the exercise: a) scenario and actor scripts; b) controller-evaluator and player handbooks, and controller-evaluator briefing/training documents; c) event controller/evaluator matrix; d) Master Scenario Event List; e) controller injects; f) Exercise Evaluation Guides for different exercise venues; g) action/problem log; h) participant feedback form and i) communication lists (see Table 3 for full material descriptions).

**Table 3:** Materials developed for the 2018 full-scale exercise in Vietnam

Materials developed	Contents	Delivered to
Controller-Evaluator Handbook	Contained all necessary tools for controllers and evaluators, including roles and responsibilities and procedures to follow during the exercise.	Controllers Evaluators
Player Handbook	Provided exercise participants with the necessary tools to perform their roles in the exercise and the procedures they should follow during the exercise. It also described exercise staff roles and responsibilities.	Players Venue Point of Contact Observers
Actors’ Script	Provided actors with biographic information of the roles they were playing, as well as timelines and details of actions that they needed to take.	Controllers Actors
Event/Controller Evaluator Matrix	Contained venue names, addresses, points of contact information, exercise staff information and hours for the exercise.	Controllers Evaluators Exercise control cell
Master Scenario Event List (MSEL)	Outlined benchmarks and injects that drove exercise play. It also included detailed input to exercise players, as well as information expected to emanate from simulated organizations. The MSEL consisted of two parts: timeline and injects.	Controllers Evaluators
Controller Injects	Described all injects for controllers. An individual event inject was a detailed description of each exercise event. There were 4 types of injects: exercise control, contextual, contingency and expected player action.	Controllers
Exercise Evaluation Guides (EEG)	Provided a consistent tool to guide data collection. It also provided a template for evaluators to write observations, accompanying discussions and recommendations and enabled them to capture strengths and areas for improvement related to each exercise objective and critical task. EEGs were designed for Commune Health Station, District Level Hospital, District Health Center, Rapid Response Team home venue, Provincial Preventive Medicine Center, Laboratory Regional Institute of Public Health, Sub-national Regional Public Health Emergency Operations Center (PHEOC) and National PHEOC.	Controllers Evaluators
Action/Problem Log	Used to record problems encountered during the exercise, particularly those that revealed potential weaknesses in the public health emergency response.	Controllers Evaluators
Participant Feedback Form	Provided participants the opportunity to comment on exercise activities and exercise design using Likert scale and open-ended questions.	Players Controllers Evaluators Venue Point of Contact Actors Observers Media Personnel
Communication List	Provided players with contact information of all venue points of contact, controllers/evaluators and some departments that players could potentially call, such as provincial hospital/ health department.	Players Controllers Evaluators Venue Point of Contact Actors Observers Media Personnel

### Conduct of the exercise

The FSX was conducted from 7 to 9 August 2018. After finalization of all materials, trainings were conducted for controllers and evaluators based in Hanoi and Quang Ninh province and actors, who were health professionals at the district hospital and commune levels. A participant briefing was also conducted at the province and district levels in Quang Ninh province. Control of the exercise was accomplished through an exercise control structure, a framework that allowed controllers to communicate and coordinate with other controllers at other exercise venues to deliver and track exercise information.

### Case scenario and process flow

The MOH sought to assess the public health infrastructure’s capacities to detect and respond to cases of severe viral pneumonia (SVP) with subsequent laboratory confirmation for MERS-CoV. Accordingly, the planning team designed a complex fictitious scenario for a 3-day FSX. Cases presenting with SVP at the district hospital and Commune Health Station (CHS), with subsequent laboratory confirmation for MERS-CoV, triggered a public health response. Four actors used scripts and symptom cards to simulate patients, family members and contacts who each had different parts of the scenario to reveal to exercise players. The agent was unknown to players, but hints were given (e.g., history of travel to Middle East) to allow case investigations to proceed toward a suspected pathogen. Actor scripts contained enough details for investigators to identify specific flights and contacts as they would in real-life investigations. However, actors were instructed to only give these details when asked, requiring players to perform thorough investigations to understand the full scenario. Actors were only active on the first day of the exercise. On the second and third days, case updates on the progress of known/suspected cases, as well as new suspected cases, were provided to the active participants, but no more in-person activity occurred at hospitals or health centers.

**Fig. 2:**
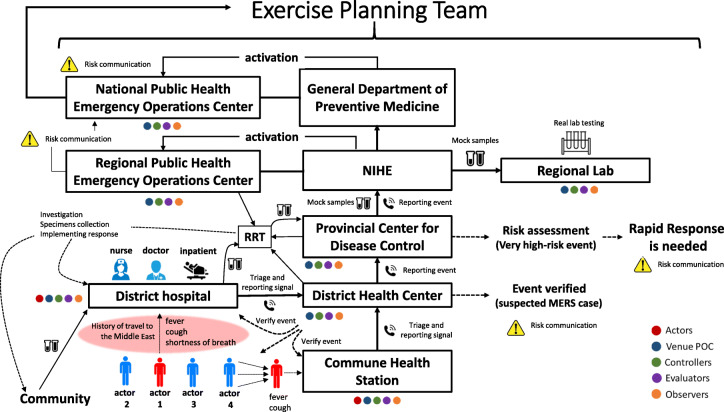
The 2018 Vietnam public health full-scale exercise scenario and process flow. The diagram provides an overview the process flow of the full-scale exercise at every administrative level. Administrative levels are shown in boxes. Solid black arrows represent interactions and flow of information/laboratory samples among different administrative levels. Dashed black arrows represent some of the main activities conducted by the administrative levels. The colored dots represent the type of players involved in each level

Throat swabs were taken from patients, but transportation to the diagnostic laboratory was simulated. Exercise controllers collected the package prepared by the hospital and prepared a similar package that contained samples “spiked” with MERS-CoV RNA (four positive and two negative) so laboratory staff could perform real tests on the exercise specimens at the regional level. Real-time polymerase chain reaction (real-time PCR) tests were conducted at the NIHE Regional Public Health Laboratory. Exercise controllers waited the anticipated time that transport would have taken, and then simulated courier delivery of the prepared package to laboratory staff so they could follow their package reception protocols. The process flow at every administrative level was also defined (Fig. 2).

### Testing the functional capacities of event-based surveillance through the FSX

Nineteen objectives and 33 critical tasks were tested by the entire FSX (Table 4). Of those, 4 objectives and 9 critical tasks corresponded to early warning and reporting, including detection, triage and verification of signals and events at the community and district levels. Detection of signals was tested at CHS and district hospital by determining if the participants recognized the case situation as corresponding to a pre-defined EBS signal. The signal triage process was tested at CHS and District Health Center (DHC) and verification was tested at CHS, the district hospital and DHC (Table 4).

**Table 4:** Critical tasks tested for the full-scale exercise 2018 in Vietnam

Critical tasks by full-scale exercise objective	Corresponding EBS steps
**Objective 1. The Commune Health Station follows established protocols to triage, verify and report signals to the District Health Center.**	• Detection • Reporting • Triage • Verification
• Signal is detected and registered • Triage is performed to determine if signal is true • If signal is true, verification is conducted • Notification is provided to Event-Based Surveillance (EBS) Focal Point at Cam Pha City Health Center	
**Objective 2. The District Hospital uses hospital signal criteria to detect and report signal.**	• Detection • Reporting
• Clinician correctly uses criteria to detect a signal in presenting patient and reports it to EBS Focal Point, who receives and logs the information	
**Objective 3. The District Hospital follows established protocols to collect specimens and package them for transport to the lab for testing.**	• Verification
• Specimens are collected and packaged for transport • PCDC is informed of when to expect specimens for testing	
**Objective 4. The District Health Center (DHC) follows established protocols to verify suspect cases and report public health events to the provincial level.**	• Triage • Verification
• Notification of signal from the hospital EBS Focal Point is received and logged • Triage performed to determine if reported signal is true. If signal is true, DHC performs a full verification at the hospital to ensure no duplication in system, registers it and reports it to Provincial Center for Disease Control (PCDC)	
**Objective 5. The District Health Center’s Rapid Response Team (RRT) deploys within timeframe requested in notification and performs public health emergency response activities according to established protocols.**	• Risk assessment
• DHC immediately deploys an RRT to hospital and commune health station • DHC’s RRT conducts case investigation • Epidemiology investigation is performed on patient’s contacts, living situation and relevant factors • DHC’s RRT provides guidance to hospital staff to isolate patient and disinfect area • Risk assessment is performed after collecting all sources of information	
**Objective 6. The PCDC follows established protocols to conduct disease surveillance, risk assessments and disease reporting in support of a public health emergency.**	• Risk assessment
• PCDC registers event reported by DHC • PCDC is in the reporting chain and monitors the public health event • Case is confirmed, risk assessment performed and overall risk is reported to National Institute of Hygiene and Epidemiology (NIHE)	
**Objective 7. The PCDC coordinates appropriate risk messaging in support of a public health emergency.**	• None
• Risk messaging is coordinated to support DHC	
**Objective 8. The PCDC’s RRT deploys within timeframe requested in notification and performs public health emergency response activities according to established protocols.**	• None
• PCDC’s RRT supports the District’s RRT activities	
**Objectives 9–19 correspond to laboratory and Public Health Emergency Operations Center (PHEOC) functions.**	• Risk assessment

### After-action review

Exercise staff conducted post-exercise “hot washes” immediately at each venue, during which participants could reflect on strengths, areas for improvement and recommendations to address shortfalls. Participants provided an overall assessment of exercise design, conduction and usefulness on a Likert scale of 1 (strongly disagree) to 5 (strongly agree) using a Participant Feedback Form. An After-Action Review (AAR) was conducted after all exercise activities were completed on 9 August to allow controllers, evaluators and stakeholders to provide an overview of simulation exercise activities and discuss strengths and areas for improvement. An AAR report and Corrective Action Plan were developed from AAR notes, feedback from supervisors’ Exercise Evaluation Guides and hot washes. The AAR report summarized key information related to the exercise and primarily focused on the analysis of capacities, including capacity performance, strengths and areas for improvement.

For this study, ethics approval was deemed unnecessary because the only research activity that involved human subjects falls into one specific exemption category as defined by the Common Rule (Code of Federal Regulations 45CFR46 Subpart A §46.104) [17]. The actors provided their verbal consent during an in-person briefing in which they received all the relevant information about the exercise and had the opportunity to ask questions about their participation.

## Results

### District hospital

The FSX began in Quang Ninh province on 7 August 2018 when a 54-year-old businessman (actor 1) presented to the emergency room of the Cam Pha city district hospital, accompanied by his wife (actor 2) and driver (actor 3) (Fig. 2). He presented with a dry cough, fatigue, muscle pain and shortness of breath. He was received by a nurse and then examined by a doctor in the emergency room. Upon questioning, he reported that 5 days prior he experienced sudden fever and cough, which had worsened over the past 2 days. As the doctor talked with him further, it was revealed that he had recently traveled to the Middle East. The patient was hospitalized *after the clinician determined he met criteria for two pre-defined signals for EBS at health facilities: a) one SVP case requiring hospital admission, and b) any suspected case of communicable diseases of group A that are required for detection and reporting according to Vietnam’s Law on Prevention and Control of Infectious Diseases* (Additional Table 1 and Additional Table 2). Upon detecting the signal, the hospital clinician reported it by phone to the Chief Medical Officer in the hospital and a nurse reported the signal to the EBS Focal Point and the director of the hospital. Following this, the hospital EBS Focal Point reported the signal to the DHC’s EBS Focal Point by phone. The time from patient arrival until signal reporting to the DHC was less than 15 min (Table 5). The following gaps were identified: the clinician did not wear a mask for the duration of his contact with the patient, nurses did not provide masks to the patient or his family members and the patient was not isolated due to lack of adequate isolation facilities.

**Table 5:** Strengths and challenges of EBS steps tested by the full-scale exercise, After-Action Review findings, Vietnam, 2018

EBS steps	FSX objectives	Strengths	Challenges
Detection and Reporting	1, 2	**Commune level** • The CHS followed established protocols to detect and report signals to the DHC in a satisfactory manner.	• The CHS lacked visible poster/communication material with the list of signals under surveillance.
		**District level (hospital)** • The hospital emergency department clinician successfully detected and reported a signal in the presenting patient.	• The hospital emergency department clinician did not use a physical listing of hospital signal criteria to detect a signal in the presenting patient.
Triage	1, 4	**Commune level** • The CHS followed established protocols to triage signals to the DHC in a satisfactory manner	• Incomplete triage by the CHS EBS Focal Point prevented certainty that the signal was true.
		**District level** • Triage by the DHC was successful	• None identified
Verification	1, 3, 4	**District level** • The DHC followed protocols to verify suspect cases and report the public health event to the provincial level	• None identified
Risk Assessment	5, 6, 9–19	**Provincial level** • The PCDC conducted a quick internal risk assessment to define response activities.	• PCDC didn’t include all available key players to conduct the risk assessment.
		**Northern Regional PHEOC** • The Northern Regional PHEOC shift from Alert mode to Response mode based on a decision-making process to assess risk.	• None identified
		**National PHEOC** The risk assessment was performed and agreed upon prior to the declaration of the emergency and activation of the PHEOC.	• None identified

Abbreviation: *CHS* Commune Health Station; *DHC* District Health Center; *EBS* Event-based surveillance; *FSX* Full-scale exercise; *PCDC* Provincial Center for Diseases Control; *PHEOC* Public Health Emergency Operations Center

### Commune Health Station

The businessman’s household employee (actor 4), who had also been feeling ill, visited the Cam Dong CHS complaining of fever, cough and shortness of breath. She was promptly attended to by the doctor; her hospital admission, her employer and his travel history were reported. The doctor identified the community-level signal *“a severe acute respiratory infection with fever in someone who has been traveling abroad in the last 2 weeks”* (Additional Table 1) and reported it to the CHS EBS Focal Point, who in turn triaged the signal. Triage was incomplete because it was not confirmed if the signal had been already reported by the hospital. The CHS EBS Focal Point reported the signal to the DHC EBS Focal Point only 22 min after the patient’s arrival and properly registered it in the logbook upon detection. The CHS lacked visible posters/communication materials displaying the list of signals under surveillance (Table 5).

### District Health Center

The district EBS Focal Point received phone calls from the district hospital and CHS reporting the signals and entered information into the district logbook. Notes from both conversations were used to inform the district RRT, which developed an immediate action plan. The district RRT was deployed to the district hospital, the CHS and the residential area of the two patients (the businessman and his household employee) and verified both signals as a single event. The RRT arrived at the district hospital within 16 min of receiving signal notification and interviewed the patient, his wife and his driver. In addition, in collaboration with the hospital laboratory team, the RRT collected throat swab specimens from the patient and his two contacts. Samples were processed following proper protocols and correctly packaged for transport to the PCDC laboratory (no real transport occurred). The RRT arrived at the CHS and patients’ residence approximately 30–40 min after notification of the signal, interviewed CHS staff and the household employee, took a throat swab from the household employee and referred her to the district hospital (simulated) for isolation and treatment. Her specimen was processed and packaged together with the district hospital samples. The pre-designed Case Investigation Form used by the RRT in the district hospital was specific for MERS-CoV. While the clinicians at the hospital correctly identified the case as SVP, activities conducted by the RRT centered on MERS-CoV as a potential pathogen before ruling out other possible causes. The DHC notified PCDC of the event and registered it in the district logbook. In addition, DHC notified the PCDC laboratory of the specimens and their estimated time of arrival.

### Provincial Center for Diseases Control

PCDC received a notification of the event from DHC, provided recommendations by phone and deployed two RRT staffers to support the district RRT: one team for the hospital and the other for the DHC (simulated). The PCDC EBS Focal Point registered the event in a logbook electronically as soon as the DHC reported it. PCDC reported the event to the regional level (NIHE and the Northern Regional PHEOC) and conducted a quick internal risk assessment to define response activities. The PCDC laboratory received samples from DHC, logged them properly and split the samples. One portion of samples was tested at PCDC (simulated) and the other portion was sent to the NIHE Regional Public Health Laboratory. The PCDC informed NIHE about the sample shipment. Early the next day, the PCDC laboratory reported to NIHE that patient #1’s sample was positive for MERS-CoV (scripted).

### National Institute of Hygiene and Epidemiology

The NIHE Regional Public Health Laboratory received samples from the PCDC laboratory (simulated). Testing using real-time PCR started about 30 min after receiving spiked samples and produced results in about 4–5 h. The NIHE Regional Public Health Laboratory tested 3 spiked samples and accurately got two positive and one negative results for MERS-CoV.

### Northern Regional Public Health Emergency Operations Center

The Northern Regional PHEOC was activated less than 1 h after receiving the report from the Quang Ninh PCDC on the verification of a MERS-CoV suspected case; however, it was delayed in informing the National PHEOC of its activation to Alert mode. The shift from Alert mode to Response mode was based on the Northern Regional PHEOC’s decision-making process to assess risk, decide on the appropriate activation level and assign PHEOC staff (Table 5). The Northern Regional PHEOC successfully activated and deployed an RRT in support of provincial public health response but experienced some communication issues when trying to connect with MOH GDPM and did not have a clear role in developing and delivering risk communications messages.

### National Public Health Emergency Operations Center

After a risk assessment was performed, an emergency was declared and the National PHEOC was activated within 30 min following standard operating procedures (SOPs) on stand-up, activation and response notification, Incident Management System (IMS) establishment and Incident Action Plan development (Table 5). Despite the above, the National PHEOC did not visibly engage in active coordination with the lower-tier response organizations within 12 h of confirmation of the public health emergency and experienced delays in communicating with the Northern Regional PHEOC. In addition, the National PHEOC lacked a centralized mechanism for tracking and reporting relevant response information such as additional updates for cases, deployment statuses and locations, resource requests and geographic mapping of cases.

### End-of-exercise evaluation

Participants positively scored the exercise training and design, giving average scores of 4.4 or above on a scale of 1 to 5 (1 strongly disagree to 5 strongly agree) ranking their agreement with positive statements about different aspects of the exercise. Data also showed that overall, participants felt that the detection and reporting arm of the surveillance system was working well with four areas for improvement: a) provide SOPs for detection and reporting at the health facility level; b) reinforce completion of the outbreak and response reports at all levels; c) reduce inconsistency of data between direct reports and manual forms at the district level; and d) improve information technology platforms generally across all levels.

## Discussion

The FSX provided an opportunity to demonstrate Vietnam’s public health response capabilities and test the EBS system and documented the value of exercising surveillance capacities as part of a real-time operational scenario before facing a true emergency. Findings of the FSX demonstrated that the EBS component of the MOH surveillance system worked well at different administrative levels. Clinicians at the District Hospital and the Commune Health Station both quickly detected a potential cluster of acute respiratory infections associated with travel to a MERS-CoV–endemic region. The cases were reported in a timely manner to EBS Focal Points at the district and province public health agencies and to the regional and national levels. Information was also reported and shared in a timely manner between different response partners at all levels.

The FSX revealed some areas for improvement. The hospitals lacked communication materials or job aids (e.g., handbooks, templates, posters) with information on signals the clinicians should be alert for and who they should notify if they encountered a public health event. Posters of signal definitions displayed more prominently would help to ensure all EBS guidelines are followed.

During the exercise, the hospital clinicians reported cases of SVP and MERS-CoV based on the patient’s history of travel and symptoms without waiting for laboratory confirmation, which is appropriate. Although not required, in doing so, clinicians and responders may have inadvertently ruled out other pathogens that would have been more common causes of SVP, such as influenza. RRT members and other responders should consider using broader forms for signal data collection prior to confirmation (i.e., using an SVP questionnaire and not one specific to MERS-CoV). It is important not to eliminate other potential causes before public health officials have a clear idea of what is happening.

Although the EBS system appeared functionally optimal, the FSX revealed that infection control, coordination with internal and external response partners and process documentation were challenging in the response and the PHEOC components of the public health system. The infection control component needed improvement, especially at the district level, where clinicians did not always adhere to best practices when using and providing personal protective equipment. Although activated alongside the PHEOC at both national and regional levels, the IMS did not always organically coordinate to ensure all aspects of the response were supported. External response partners, such as CDC and WHO VTN, could have coordinated more fully in risk assessments, response activities, risk communication and during meetings. Much of this miscommunication was driven by lack of clarity on roles and responsibilities between administrative levels. Although timely reporting of information was a strength at multiple levels, managing, displaying and coordinating information within PHEOCs was more challenging. Most PHEOCs would benefit from a concerted effort to develop and support a Common Operational Picture.

Overall, exercise participants performed professionally and had a good understanding of their jobs. However, the lack of process documentation tools, such as IMS forms, SOPs and job aids hindered optimal staff performance at all levels. Some SOPs and templates have been developed, but they require further refinement and dissemination and potential users/stakeholders throughout the response system need to be trained on them. Documentation of roles, responsibilities, authorization and processes also helps ensure continuity of operations, even if key personnel are missing during a real emergency. Although the district-level RRTs performed optimally, the FSX showed that the next step for RRTs at all levels is to codify successes and lessons learned into a robust set of SOPs, supported by national guidelines and training materials for RRT establishment and operations at different administrative levels. Although we did not evaluate transportation of respiratory samples from health facilities to the province and regional laboratory, it is important to recognize that transport is often a chokepoint for a timely response.

Evaluating an EBS system is challenging [2, 3]. We previously evaluated EBS systems in Vietnam using mixed methods that included 1) a retrospective data collection table sent electronically to all districts to collect logbook time stamps for event notification and response, 2) questionnaires sent electronically to all levels with acceptability and sustainability related questions and 3) key informant interviews and focus group discussions through field visits [10, 11, 16]. Evaluations showed that the pilot EBS worked as an effective early warning system for Vietnam. This FSX demonstrated that EBS enables the rapid detection and immediate notification of emerging public health threats. However, it is important to recognize that, although we tried not to disclose the identity of the pathogen from the participants, we were not able to keep them from knowing when the exercise would take place. So, participants were more likely to report “something” that might be overlooked in day-to-day situations. For this reason, exercise findings suggesting the EBS system would perform in the real world as it did during the exercise need to be interpreted with caution. Conducting exercises without participants prior knowledge should be considered.

Preparing and conducting an FSX is time and resource consuming. The planning process for this particular exercise took 9–12 months and included representatives from all exercise components on the planning committee. It is necessary to coordinate with all participating entities from the beginning, as well as to identify, recruit and train players, evaluators and controllers. Finally, it is essential to implement efficient logistical support for a successful operational performance such as procurement and distribution of catering, invitations, venues setup, facilitator and participant accommodation and transport, supplies, equipment, print/documents and translations services.

Among the lists of EBS signals, “respiratory infection with fever in someone who has been traveling abroad in the last 14 days” was an important one that helped detect imported cases of COVID-19 and their contacts in the community, at borders and in hospitals. Learning from the experience of implementing event-based surveillance, the MOH Vietnam developed and disseminated different types of communication materials, including posters, flyers and video clips for community level education for early detection and immediate reporting of COVID-19 cases. These early measures may have ensured rapid detection and reporting of signals for COVID-19 and efficient contact tracing of SARS-CoV-2 confirmed cases, thus minimizing community transmission in Vietnam [18]. Additionally, EBS in health care facilities has been enhanced with physicians in all hospitals and clinics sensitized to detect and report COVID-19 specific signals. Understanding the key role EBS can play during the COVID-19 pandemic, the MOH is strengthening and expanding EBS in country and including it in its guidance and recommendations for COVID-19 surveillance and response measures.

By describing the design and the process of the FSX in this manuscript, we hope that other Ministries of Health, non-governmental organizations and public health-related agencies can use similar approaches to test functionalities of their EBS, laboratory, RRT and PHEOC systems to improve early warning and response systems, as Vietnam has done and continues to do.

## Conclusions

This exercise documents the value of exercising surveillance capabilities as part of a real-time operational scenario before facing a true emergency. The timing of this exercise and choice of disease scenario was particularly fortuitous given the subsequent appearance of COVID-19. As a result of this exercise and subsequent improvements made by the MOH, the country may have been better able to deal with the emergence of SARS-CoV-2 and contain it.

## Supplementary Information

**Additional file 1: Table S1.** List of signals for event-based surveillance at community-level and health facilities in Vietnam. This file contains the list of predefined signals to implement event-based surveillance in communities and health facilities in Vietnam.

**Additional file 2: Table S2.** List of communicable diseases to be reported in Vietnam. This file contains the current list of communicable diseases to be reported by the surveillance system in Vietnam.

## Data Availability

The authors declare that the data supporting the findings of this study are available within the article and its additional information files.

## Abbreviations

AAR: After-action review
CDC: U.S. Centers for Disease Control and Prevention
CHS: Commune Health Station
DHC: District Health Center
EBS: Event-based surveillance
EEG: Exercise evaluation guides
FSX: Full-scale exercise
GDPM: General Department of Preventive Medicine
GHSA: Global Health Security Agenda
IAP: Incident action plan
IHR: International Health Regulations 2005
IMS: Incident management system
MERS-CoV: Middle East Respiratory Syndrome–Coronavirus
MOH: Ministry of Health
MSEL: Master scenario event list
NIHE: National Institute of Hygiene and Epidemiology
PCDC: Provincial Center for Diseases Control
PCR: Polymerase Chain Reaction
PHEOC: Public Health Emergency Operations Center
RRT: Rapid response team
SOP: Standard operating procedure
SVP: Severe viral pneumonia
WHO: World Health Organization
WHO VTN: World Health Organization office in Vietnam
