# Supplementary material for: Testing early warning and response systems through a full-scale exercise in Vietnam
Source: BMC Public Health. 2021 Feb 26;21:409. doi: 10.1186/s12889-021-10402-x (PMC7907319; doi:10.1186/s12889-021-10402-x)
Supplement: Supplementary file 1 — Additional file 1: Table S1. List of signals for event-based surveillance at community-level and health facilities in Vietnam. This file contains the list of predefined signals to implement event-based surveillance in communities and health facilities in Vietnam. [file 12889_2021_10402_MOESM1_ESM.pdf]

Additional table 1. List of signals for event-based surveillance at community-level and health facilities in Vietnam.

For community level	For health facilities
1. A child less than 15 years old with sudden weakness of limbs	1. Healthcare workers with severe illness requiring hospital admission or resulting in death, after caring for patients with similar symptoms.<br>2. Two or more cases of severe acute respiratory infections within 7 days in the same community, household, school, or workplace.<br>3. One case of severe viral pneumonia requiring hospital admission.<br>4. Unexpectedly large increase of cases of the same symptoms, based on clinician's professional judgements.<br>5. Two or more cases of infectious diseases with the same symptoms from the same location (e.g. household, residential unit, school, factory).<br>6. One case of malaria in an area where the disease has been eliminated or never circulated before.<br>7. Occurrence of unexplained or unusual clinical manifestation or treatment response of a known infectious disease based on clinician's professional judgements.<br>8. Occurrence of one or more cases or deaths of a strange, unusual or unexplained disease, based on clinician's professional judgments.<br>9. Unexpected increase of people being vaccinated for rabies in the same community.<br>10. Any suspected cases of communicable diseases of group A according to the Law on Prevention and Control of Infectious Diseases (2007).
2. A single case with fever and rash, accompanied by cough or pink eyes	
3. A single case that is severe enough to require hospital admission or dies with any of the followings:<br>a) Three or more rice watery stools within 24 hours in any person 5 years old or older with dehydration.<br>b) Respiratory infection with fever in someone who has been traveling abroad in the last 14 days.<br>c) Respiratory infection with fever after contact with live poultry in the last 14 days.<br>d) Illness within 7 days following vaccination.<br>e) Illness which has never been seen before, or with rare symptoms, in the community.<br>f) An unexplained death.	
4. Two or more hospitalized cases and/or death(s) with similar symptoms occurring in the same community, school, or workplace within 7 days.	
5. Unusual large numbers of one of the followings:<br>a) Children absent from the same school due to the same illness within 7 days.<br>b) People buying medicines for fever, cough, or diarrhea at pharmacies in the same residential area within 1 week.<br>c) People sick with similar types of symptoms at the same time.<br>d) Sickness or die-off of poultry, domestic animals, or other animals.	
6. Any dog that:<br>a) Is suspected as a rabid dog<br>b) Is sick and has bitten someone<br>c) Has bitten two or more people in the last 10 days.	
