# Supplementary material for: Testing early warning and response systems through a full-scale exercise in Vietnam
Source: BMC Public Health. 2021 Feb 26;21:409. doi: 10.1186/s12889-021-10402-x (PMC7907319; doi:10.1186/s12889-021-10402-x)
Supplement: Supplementary file 2 — Additional file 2: Table S2. List of communicable diseases to be reported in Vietnam. This file contains the current list of communicable diseases to be reported by the surveillance system in Vietnam. [file 12889_2021_10402_MOESM2_ESM.pdf]

Additional table 2. List of communicable diseases to be reported in Vietnam (Promulgated under Circular No. 54/2015/TT- BYT dated 28 December 2015).

1. Dangerous communicable diseases for which each individual case must be reported immediately after diagnosis and no later than 24 hours

No.	Name of diseases	Group	ICD10 codes
1	Poliomyelitis	A	A80
2	Diphtheria	B	A36
3	Streptococcus suis in humans	B	B95
4	Avian influenza A(H5N1)	A	J10/A(H5N1)
5	Avian influenza A(H7N9)	A	J10/A(H7N9)
6	Plague	A	A20
7	Ebola	A	A98.4
8	Lassa	A	A96.2
9	Marburg	A	A98.3
10	Rubella	B	B06
11	West Nile fever	A	A 92.3
12	Yellow fever	A	A95
13	Dengue hemorrhagic fever	B	A91
14	Measles	B	B05
15	Cholera	A	A00
16	Hand, foot, and mouth disease	B	A08.4
17	Anthrax	B	A22
18	Middle East Respiratory Syndrome caused by coronavirus (MERS-CoV)	A	
19	Meningococcal meningitis	B	A39.0
20	Dangerous emerging infectious diseases and new diseases of unknown causes	A	

2. Dangerous communicable diseases for which each individual case must be reported after diagnosis and no later than 48 hours

No.	Name of diseases	Group	ICD10 codes
21	Rabies	B	A82
22	Whooping-cough (pertussis)	B	A37
23	Acute flaccid paralysis with suspected poliomyelitis		
24	Pulmonary tuberculosis	B	A15
25	Malaria	B	B50 - B54
26	Typhoid	B	A01
27	Neonatal tetanus	B	A33
28	Other tetanus	B	A34, A35
29	Viral Hepatitis A	B	B15
30	Viral Hepatitis B	B	B16
31	Viral Hepatitis C	B	B17.1
32	Japanese encephalitis	B	A83.0
33	Other viral encephalitis	B	A83 - A85
34	Leptospirosis	B	A27

3. Communicable diseases for which number of cases and deaths must be reported on a monthly basis

No.	Name of diseases	Group	ICD10 codes
35	Adenovirus disease	B	B30.0 - B30.3
36	Influenza	B	J10
37	Amoebic dysentery	B	A06
38	Bacillary dysentery	B	A03
39	Parotitis (mumps)	B	B26
40	Chickenpox	B	B01
41	Diarrhea	B	A09
42	Other viral hepatitis (or without virus typing)		
